# Associations of the Healthy Eating Index-2010 with risk of all-cause and heart disease mortality among adults with hypertension: Results from the National Health and Nutrition Examination Survey 2007–2014

**DOI:** 10.3389/fnut.2023.1077896

**Published:** 2023-03-03

**Authors:** Yuhui Zhang, Duanbin Li, Haizhu Zhang

**Affiliations:** ^1^Department of Cardiology, Anyang People's Hospital, Xinxiang Medical University, Anyang, China; ^2^Department of Cardiology, Sir Run Shaw Hospital, College of Medicine, Zhejiang University, Hangzhou, China

**Keywords:** Healthy Eating Index, adults with hypertension, heart disease, mortality, NHANES

## Abstract

**Background:**

Studies regarding the impact of the Healthy Eating Index-2010 (HEI-2010) on the mortality of adults with hypertension are lacking.

**Objectives:**

This study aimed to prospectively explore the relationships between HEI-2010 and mortality from heart disease and all causes in adults with hypertension based on the National Health and Nutrition Examination Survey (NHANES), 2007–2014.

**Methods:**

This is a prospective cohort study including 6,690 adults with hypertension from NHANES (2007–2014). National Death Index data up to 31 December 2019 were used to determine the number of deaths due to heart disease and all other causes. We evaluated hazard ratios (HRs) and 95% confidence intervals (CIs) using the Cox proportional hazards model.

**Results:**

A total of 1,259 deaths from all causes, including 338 due to heart disease, were documented over an average follow-up duration of 8.4 years. In comparison with the lowest quartile of HEI-2010 scores, multivariable-adjusted HRs (95% CIs) for all-cause mortality were 0.82 (0.70, 0.97), 0.78 (0.64, 0.95), and 0.68 (0.54, 0.85) for the second, third, and fourth quartiles of the HEI-2010 scores (*P*-trend < 0.001) and for heart disease mortality were 0.60 (0.44, 0.81), 0.59 (0.40, 0.89), and 0.53 (0.35, 0.80) (*P*-trend = 0.010). Each increment in natural-log-transformed HEI-2010 scores was linked to a 43% reduction in the risk of all-cause mortality (*P* < 0.001) and a 55% reduction in the risk of heart disease mortality (*P* = 0.003). Among the 12 components of HEI-2010, adherence to a higher intake of greens and beans, vegetables, total protein foods, seafood and plant proteins, and unsaturated fatty acids, as well as moderate consumption of empty calories, were related to a 21–29% lower risk of all-cause mortality.

**Conclusion:**

In the current study, there was a statistically significant inverse relationship between HEI-2010 and mortality from heart disease and all causes among adults with hypertension. Based on the findings, it may help guide the dietary intake for adults with hypertension.

## 1. Introduction

Hypertension is becoming a global public health challenge. Among people aged 30–79 years, the prevalence of hypertension doubled from 1990 to 2019 (1). Elevated blood pressure (BP) has been recognized to be responsible for pathophysiological changes in the end organs of the brain (infarction and hemorrhage), the heart (myocardial ischemia and left ventricular hypertrophy, and heart failure), and the kidneys (renal sclerosis and proteinuria) (2). The presence of hypertension increases the risk of cardiovascular disease (CVD) and stroke, leading to an increase in CVD and all-cause mortality (3, 4). With the growing recognition of the major role diet plays in disease risk, identifying healthy dietary patterns that can prevent CVD and premature death in adults with hypertension is critical.

Dietary research is increasingly focusing on dietary patterns rather than single nutrients or food groups as dietary components are interrelated and consumed in combination. Healthy dietary patterns were characterized as diets low in saturated fat, added sugars, and sodium and high in vegetables, whole grains, fruits, lean protein, and low- and non-fat dairy (5). The HEI, as one of the healthy dietary patterns, is a comprehensive measurement of dietary quality in line with the Dietary Guidelines for Americans (DGA) and is the basis for US government nutrition policy. Based on the DGA recommendations, which included adding fruits, vegetables, low-fat dairy products, and whole grains, as well as limiting added sugars, saturated fats, and refined grains, the HEI generated scores for each component and a total score showing the diet quality over multiple dietary dimensions (6). Modeled on the 2010 DGA recommendations, the HEI-2010 included 12 components that were proven to be a reliable and valid measurement of dietary quality for Americans (7). In two studies, HEI-2010 has been shown to have a reverse relationship with serum C-reactive protein (CRP), apolipoprotein B, and systolic blood pressure (8, 9). In a cross-sectional research study involving 1036 women in Iran, the HEI-2010 was related to a lower metabolic syndrome risk (10). Meanwhile, two other prospective cohort studies suggested that HEI-2010 was inversely correlated with CVD and all-cause mortality in multiethnic populations (11) or older adults (12). Even with these advantages, among individuals with hypertension who often had unhealthy lifestyle factors (13), endothelial dysfunction, increased oxidative stress, vascular remodeling (14), pro-inflammatory release (15), and higher risk of developing heart disease and mortality, the health impacts of HEI-2010 on heart disease mortality remain unclear.

Therefore, the purposes of the present study were to explore the relationship of HEI-2010 and its components with mortality from heart disease and all causes in adults with hypertension based on NHANES.

## 2. Materials and methods

### 2.1. Study population

With a nationally representative sample, NHANES estimates the nutritional and health status of the US civilian population. The survey was performed by the National Center for Health Statistics, and it collected information through personal structured interviews at home, health screenings at a mobile examination center (MEC), and laboratory examinations. Detailed information can be obtained elsewhere (https://www.cdc.gov/nchs/nhanes/index.htm).

Based on NHANES 2007–2014 surveys, 7914 participants with hypertension aged 20 years and older were included when complete dietary intakes of the 2-day dietary interviews were provided. Hypertension was defined as systolic blood pressure (SBP) ≥140 mmHg and/or diastolic blood pressure (DBP) ≥ 90 mmHg, physician-diagnosed hypertension, or consuming anti-hypertensive medicine. In the final analysis, 6,690 participants with hypertension were included after excluding those who reported pregnancy (*n*=20), cancer (*n* = 1,200) or those with no follow-up data (*n* = 4) (Supplementary Figure 1).

### 2.2. HEI-2010 scores

HEI-2010 was calculated to indicate diet quality from two 24-h dietary recollection data collected, one of which was a face-to-face survey conducted at the MEC by trained interviewers, followed by a telephone follow-up 3–10 days later to obtain a more complete picture of the usual dietary intake of the US population. Average dietary intake data were used for analysis only when participants completed two 24-h recalls. A total of 12 components make up HEI-2010, with nine adequacy components (total vegetables, whole fruits, total fruits, whole grains, greens and beans, total dairy, seafood and plant proteins, total protein foods, and fatty acid ratio) and three moderation components (sodium, empty calories, and refined grains). Supplementary Table 1 illustrates HEI-2010 components along with their point values and scoring criteria (6). The HEI 2010 scores consist of 12 dietary component scores, which add up to a total score of 100. For adequacy components, the highest score was awarded for intake at or above the criteria. As for the moderation components, the highest score was awarded for intake at or below the criteria. A proportional score is assigned to intakes between the lowest and highest criteria (6). For each of the 12 HEI-2010 components, participants were considered compliant if they obtained the highest component score; otherwise, they were classified as non-compliant. Thus, the compliant participants had the highest score of 5, 10, or 20, while non-compliant participants scored lower than this, with higher scores indicating closer adherence to the dietary guidelines.

### 2.3. Ascertainment of mortality

We assessed mortality over the follow-up period based on National Death Index records through 31 December 2019 (https://www.cdc.gov/nchs/data-linkage/mortality-public.htm). The codes of the *International Classification of Diseases, Tenth Revision* (*ICD-10*) were used to classify the causes of death. Death due to heart disease included rheumatic heart disease, hypertensive heart and renal disease, ischemic heart disease, and heart failure (ICD10 codes I00–I09, I11, I13, and I20–I51). Other causes of death included chronic lower respiratory diseases (J40–J47), malignant neoplasms (C00–C97), cerebrovascular diseases (I60–I69), influenza and pneumonia (J09–J18), Alzheimer's disease (G30), diabetes mellitus (E10–E14), nephritis, nephrotic syndrome and nephrosis (N00–N07, N17–N19, N25–N27), accidents (unintentional injuries) (V01–X59, Y85–Y86), and all other causes (residual).

### 2.4. Assessment of covariates

Sociodemographic variables included age, sex (male and female), ethnicity (non-Hispanic white, non-Hispanic Black, Mexican American, and other races), and education (below high school, high school, and above high school), which were assessed during the interview. Corresponding questionnaires and MEC obtained data on body mass index (BMI), alcohol consumption, smoking, dietary intake, recreational activity, anti-hypertensive medicine use, blood pressure level, and presence of hyperlipidemia, diabetes, and CVD. Never smokers were defined as those who smoked <100 cigarettes in their lifetime. Those who smoked >100 cigarettes and no longer smoke were considered former smokers, and those who smoked >100 cigarettes in their lifetime and still smoke some days or every day were considered current smokers. Drinking status was grouped into nondrinker, low-to-moderate drinker (<3 drinks/day in men and <2 drinks/day in women), or heavy drinker (≥3 drinks/day in men and ≥2 drinks/day in women). The recreational activity was categorized into three groups: inactive (no recreational physical activity), moderately active (moderate recreational physical activity), or vigorously active (vigorous recreational physical activity). Based on the physical activity questionnaire, recreational activities leading to a slight increase in heart rate or breath such as bicycling, brisk walking, golf, or swimming for at least 10 continuous min were considered moderate activity, and recreational activities leading to great increases in heart rate or breath such as running or basketball for at least 10 continuous min were considered vigorous activity.

Diabetes is defined as fasting blood glucose ≥7.0 mmol/L, plasma glucose levels 2 h after meals ≥11.1 mmol/L, physician-diagnosed diabetes, use of hypoglycemic medications, or glycosylated hemoglobin (HbA1c) ≥6.5% (16). Participants with total cholesterol levels ≥240 mg/dL, fasting triglyceride ≥150 mg/dL, high-density lipoprotein (HDL) cholesterol <40 mg/dL, low-density lipoprotein (LDL) cholesterol ≥100 mg/dL, and a history of taking lipid-lowering medications were regarded as hyperlipidemia. Based on self-reported information, CVD (yes/no) and anti-hypertensive medicine use (yes/no) were defined. Three and sometimes four BP determinations (systolic and diastolic) are taken in the MEC and during home examinations on all eligible individuals using a mercury sphygmomanometer. SBP average and DBP average represent blood pressure results that were reported to the examinee. A detailed information can be obtained elsewhere (https://wwwn.cdc.gov/Nchs/Nhanes/2001-2002/BPX_B.htm#Quality_Assurance_&_Quality_Control). In our study, the SBP average and DBP average represent blood pressure results. We categorized participants into two groups based on their blood pressure levels: SBP/DBP <160/100 mmHg (either SBP <160 mmHg and/or DBP <100 mmHg) and SBP/DBP ≥ 160/100 mmHg (either SBP ≥ 160 mmHg and/or DBP ≥ 100 mmHg). Furthermore, strict laboratory analyses, including measurement of estimated glomerular filtration rate (eGFR) and total cholesterol levels at baseline, were conducted.

### 2.5. Statistical analysis

All analyses were conducted using sample weights, strata, and primary sampling units to obtain accurate national estimates. Continuous variables were expressed as mean (SE) and categorical variables as numbers (percentages). HEI-2010 total scores were divided into quartiles, and the difference between the four groups was compared by one-way ANOVA tests (continuous variables with normal distribution) and χ^2^ test (categorical variables). We estimated HRs and 95% CIs for heart disease and all-cause mortality based on quartiles of HEI-2010 scores using the Cox proportional hazards model. Person-time is referred to the period between the NHANES interview date and the date of death or the end of the follow-up (31 December 2019). We fitted three statistical models. Model 1 was adjusted for age (continuous), sex (male or female), and ethnicity (non-Hispanic white, non-Hispanic Black, Mexican American, and other race). Model 2 was adjusted for education (below high school, high school, and above high school), BMI (continuous), smoking status (never smoker, former smoker, and current smoker), drinking status (nondrinker, low-to-moderate drinker, and heavy drinker), recreational activity (inactive, moderately active, and vigorously active), and total energy intakes (in quartiles). Model 3 was further adjusted for blood pressure level (SBP/DBP ≥ 160/100 mmHg or SBP/DBP <160/100 mmHg), anti-hypertensive medicine use (yes or no), hyperlipidemia (yes or no), diabetes (yes or no), and CVD (yes or no). To analyze the linear trend, each category was assigned a median value as a continuous variable. Multiple imputations were conducted to minimize the reduction in sample size resulting from missing covariates.

To investigate dose-response associations between HEI-2010 scores and mortality, we used a restricted cubic spline regression model with four knots at the 5th, 35th, 65th, and 95th percentiles of the HEI-2010 scores, excluding the most extreme 5% values to reduce the potential influence of outliers. The likelihood ratio test was used for testing non-linearity. Furthermore, stratified analyses were performed to assess whether the relationship of HEI-2010 scores with all-cause mortality differed by age (<60 and ≥60 years), sex (men and women), ethnicity (non-Hispanic white and others), BMI (<30 and ≥30), drinking status (non-drinker and drinker), smoking status (never smoker and former/current smoker), recreational activity (inactive group and active group), blood pressure level (SBP/DBP ≥ 160/100 mmHg or SBP/DBP <160/100 mmHg), anti-hypertensive medicine use (yes or no), hyperlipidemia (yes or no), diabetes (yes or no), and CVD (yes or no). The *P*-value of the product term between continuous HEI-2010 scores and stratified variables was calculated to assess the significance of the interaction.

To determine whether statistically significant correlations were ascribed to specific components of the HEI-2010, we further assessed the correlation between the HEI-2010 components and all-cause mortality adjusted for all covariates. We classified the component scores by selecting appropriate cutoff points according to the overall sample score distribution or restricted cubic spline model. To better show the percentage contribution of the dietary components to the maximum possible score, we created radar plots.

We also conducted several sensitivity analyses to assess the robustness of our findings. First, we excluded participants who died within the first 2 years of follow-up (*n* = 199) to minimize the potential reverse causation bias. Second, we further adjusted for individual foods or nutrients, including intakes of fiber, total fat, cholesterol, vitamin A, vitamin C, and vitamin E (all in quartiles). Third, we further adjusted for other biomarkers, including total cholesterol levels and eGFR (all in quartiles). Finally, we also assessed the associations of HEI-2010 scores with cerebrovascular deaths and cancer deaths.

We performed all analyses with R version 4.2.0. A two-sided *P*-value of < 0.05 was considered statistically significant.

## 3. Results

### 3.1. Participants characteristics

The baseline characteristics according to quartiles of HEI-2010 scores are summarized in Table 1. Out of 6,690 participants with hypertension (mean age, 55.91 years; 51.9% women), the median (interquartile range) HEI-2010 score was 53.9 (44.1, 64.1). Individuals with higher HEI-2010 scores were older, were more likely to be women, tended to have higher educational levels, engaged in more recreational activities, never smoked, and were less likely to be obese.

**Table 1:** Study characteristics of participants with hypertension in NHANES 2007–2014 according to HEI-2010 scores quartiles (*n* = 6,690)^a^.

**Characteristics**	**HEI-2010 scores**					
	**Total**					***P*** **value**
Range		14.0–44.1	44.1–53.9	53.9–64.1	64.1–98.8	
Patients, *n*	6,690	1,668	1,682	1,667	1,673	
Age, years	55.91 ± 0.25	50.61 ± 0.45	55.36 ± 0.52	57.89 ± 0.48	59.93 ± 0.42	<0.001
Sex, *n* (%)						<0.001
Women	3,471 (51.90)	751 (45.03)	851 (48.92)	881 (51.96)	988 (59.87)	
Men	3,219 (48.10)	917 (54.97)	831 (51.08)	786 (48.04)	685 (40.13)	
Ethnicity, *n* (%)						<0.001
Non-Hispanic white	2,922 (43.70)	762 (66.41)	769 (70.30)	690 (69.51)	701 (69.18)	
Non-Hispanic Black	1,862 (27.80)	535 (18.61)	474 (15.07)	449 (13.93)	404 (12.43)	
Mexican American	824 (12.30)	185 (6.53)	191 (5.59)	258 (7.30)	190 (5.10)	
Other race	1,082 (16.20)	186 (8.45)	248 (9.05)	270 (9.26)	378 (13.28)	
BMI, kg m^2^	31.18 ± 0.13	32.08 ± 0.19	31.50 ± 0.27	31.12 ± 0.22	29.95 ± 0.19	<0.001
Education, n (%)						<0.001
Below high school	1,926 (28.80)	538 (23.75)	493 (20.63)	499 (20.29)	396 (15.28)	
High school	1,685 (25.20)	478 (30.00)	461 (27.72)	405 (24.08)	341 (19.96)	
Above high school	3,079 (46.00)	652 (46.25)	728 (51.65)	763 (55.63)	936 (64.76)	
Recreational activity, *n* (%)						<0.001
Inactive	4,060 (60.70)	1,142 (64.72)	1,077 (58.82)	997 (54.90)	844 (45.90)	
Moderately active	1,819 (27.20)	371 (25.68)	425 (28.68)	462 (30.26)	561 (34.10)	
Vigorously active	811 (12.10)	155 (9.60)	180 (12.50)	208 (14.84)	268 (20.00)	
Smoking status, *n* (%)						<0.001
Never smoker	3,452 (51.60)	708 (45.40)	825 (49.85)	909 (53.09)	1,010 (57.65)	
Former smoker	1,966 (29.40)	420 (23.30)	494 (29.50)	529 (33.43)	523 (33.87)	
Current smoker	1,272 (19.00)	540 (31.29)	363 (20.65)	229 (13.48)	140 (8.48)	
Drinking status, *n* (%)						<0.001
Non-drinker	2,671 (39.90)	650 (35.02)	639 (31.44)	686 (32.50)	696 (33.49)	
Low-to-moderate drinker	2,996 (44.80)	646 (41.91)	741 (49.40)	789 (54.82)	820 (57.53)	
Heavy drinker	1,023 (15.30)	372 (23.07)	302 (19.16)	192 (12.68)	157 (8.97)	
Total energy intakes, *n*						<0.001
Q1 (<2,702)	1,673 (25.00)	349 (15.45)	381 (17.67)	446 (21.24)	497 (24.43)	
Q2 (2,702–3,591)	1,671 (25.00)	332 (19.24)	421 (25.33)	449 (26.13)	469 (27.20)	
Q3 (3,591–4,644)	1,673 (25.00)	413 (26.67)	441 (26.06)	397 (26.23)	422 (28.66)	
Q4 (≥4,644)	1,673 (25.00)	574 (38.64)	439 (30.94)	375 (26.39)	285 (19.72)	
Blood pressure level, *n* (%)						0.630
SBP/DBP <160/100 mmHg	5,976 (89.30)	1,493 (90.97)	1,511 (91.64)	1,469 (90.48)	1,503 (91.64)	
SBP/DBP ≥ 160/100 mmHg	714 (10.70)	175 (9.03)	171 (8.36)	198 (9.52)	170 (8.36)	
Anti-hypertensive medicine use, *n* (%)						0.100
No	5,579 (83.40)	1,428 (86.04)	1,402 (83.99)	1,374 (83.50)	1,375 (81.77)	
Yes	1,111 (16.60)	240 (13.96)	280 (16.01)	293 (16.50)	298 (18.23)	
CVD, *n* (%)	1,280 (19.10)	330 (15.68)	315 (15.95)	340 (17.69)	295 (15.82)	0.580
Diabetes, *n* (%)	2,079 (31.10)	441 (21.39)	503 (24.60)	589 (27.50)	546 (25.72)	0.010
Hyperlipidemia, *n* (%)	5,359 (80.10)	1,322 (81.09)	1,321 (78.73)	1,379 (83.82)	1,337 (80.62)	0.080

a Continuous variables are described as mean (SE). Categorical variables are presented as numbers (percentages). Complex survey designs were considered in all estimates. BMI, body mass index; CVD, cardiovascular disease; Q, quartile; NHANES, National Health and Nutrition Examination Surveys.

### 3.2. HEI-2010 scores and mortality

**Figure 1:**
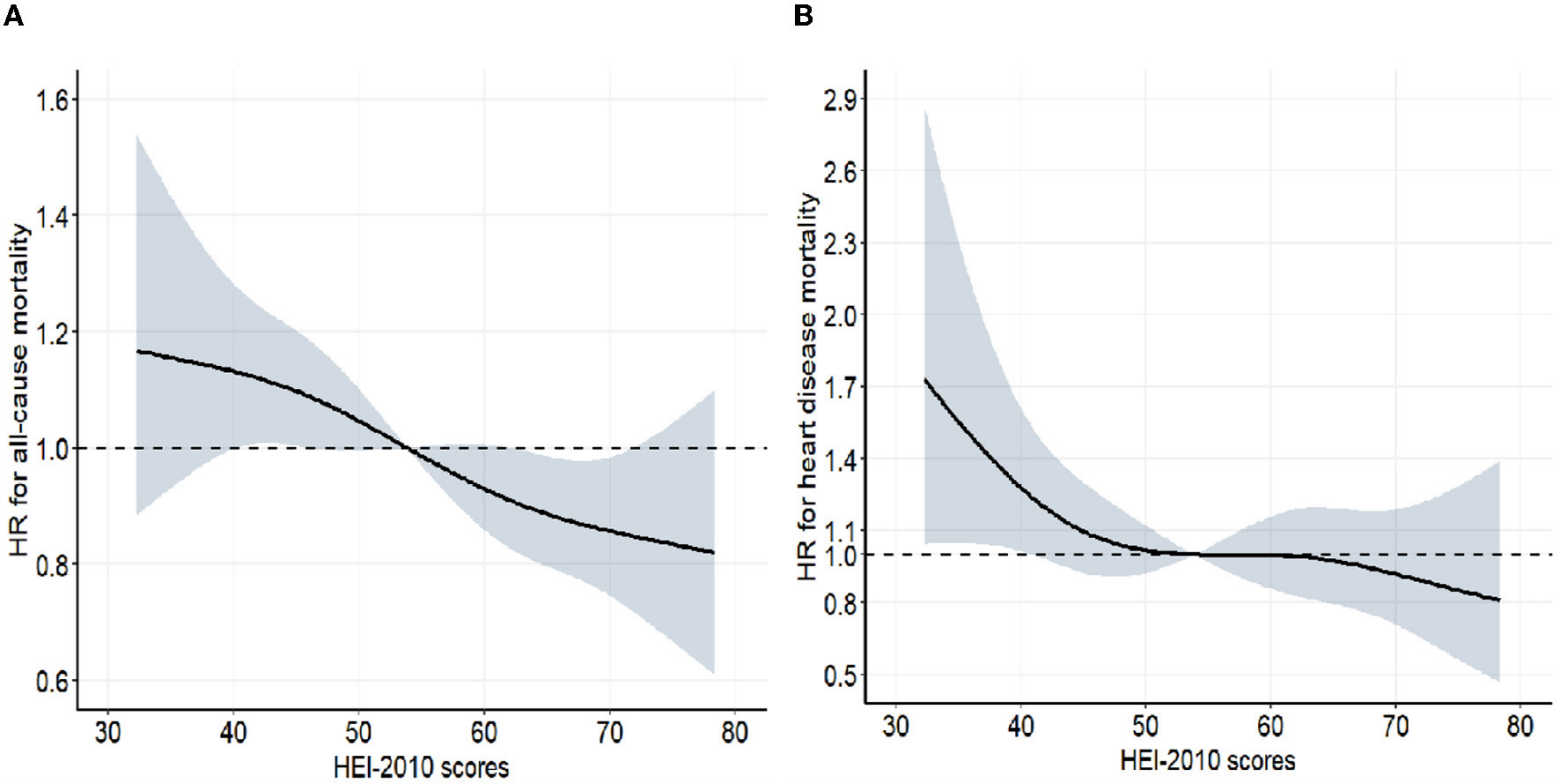
Associations between HEI 2010 and mortality from all-cause **(A)** and heart disease **(B)** among participants with hypertension from NHANES 2007–2014 (*n* = 6,690). The dose–response relationships between HEI 2010 scores and all-cause and heart disease mortality were assessed by a restricted cubic spline regression model with four knots (5th, 35th, 65th, and 95th percentiles) adjusted for age (continuous), sex (men or women), ethnicity (non-Hispanic white, non-Hispanic Black, Mexican American, and other race), education (below high school, high school, and above high school), BMI (continuous), smoking status (never smoker, former smoker, and current smoker), drinking status (non-drinker, low-to-moderate drinker, and heavy drinker), recreational activity (inactive, moderately active, and vigorously active), total energy intakes (in quartiles), blood pressure level (SBP/DBP ≥ 160/100 mmHg or SBP/DBP <160/100 mmHg), anti-hypertensive medicine use (yes or no), hyperlipidemia (yes or no), diabetes (yes or no), and CVD (yes or no). Gray-shaded areas represent 95% confidence intervals. For all-cause mortality, *P*-linearity was <0.001, and for heart disease mortality, 0.010.

During an average follow-up of 8.4 years, 1259 deaths from all causes were documented, including 338 heart disease deaths. The relationship between HEI-2010 scores with heart disease and all-cause mortality is presented in Table 2. After multivariate adjustment, higher HEI-2010 scores were linked to lower heart disease and all-cause mortality. In comparison with the lowest quartile of HEI-2010 scores, multivariable-adjusted HRs (95% CIs) for all-cause mortality were 0.82 (0.70, 0.97), 0.78 (0.64, 0.95), and 0.68 (0.54, 0.85) for the second, third, and fourth quartiles of the HEI-2010 scores (*P*-trend < 0.001), as for heart disease mortality were 0.60 (0.44, 0.81), 0.59 (0.40, 0.89), and 0.53 (0.35, 0.80) (*P*-trend = 0.010). The curve associations of HEI-2010 scores (range: 32.2–78.6) with all-cause mortality (non-linear *P* = 0.899) and heart disease mortality (non-linear *P* = 0.455) were described based on the restricted cubic spline models (Figure 1). Each increment in natural-log-transformed HEI-2010 scores was linked to a 43% reduction in the risk of all-cause mortality (*P* < 0.001) and a 55% reduction in the risk of heart disease mortality (*P* = 0.003).

**Table 2:** HR of all-cause and heart disease mortality according to quartiles of HEI-2010 scores among participants with hypertension in NHANES 2007–2014.

**Characteristic**	**HEI-2010 scores HR (95% CI)**					
	**Q1**	**Q2**	**Q3**	**Q4**	***P*** **-trend**	**Per-unit increment in ln-transformed HEI-2010 scores**
**Range**	14.0–44.1	44.1–53.9	53.9–64.1	64.1–98.8		
**All-cause mortality**						
No. of deaths/total	320/1,668	329/1,682	323/1,667	287/1,673		
Model 1^a^	1.00	0.72 (0.61, 0.86)	0.63 (0.52, 0.76)	0.51 (0.41, 0.63)	<0.001	0.38 (0.29, 0.50) <0.001
Model 2^b^	1.00	0.82 (0.70, 0.97)	0.80 (0.64, 0.95)	0.70 (0.56, 0.87)	0.002	0.58 (0.43, 0.79) <0.001
Model 3^c^	1.00	0.82 (0.70, 0.97)	0.78 (0.64, 0.95)	0.68 (0.54, 0.85)	<0.001	0.57 (0.42, 0.77) <0.001
**Heart disease mortality**						
No. of deaths/total	85/1,668	83/1,682	86/1,667	84/1,673		
Model 1^a^	1.00	0.54 (0.39, 0.75)	0.52 (0.36, 0.75)	0.46 (0.30, 0.70)	0.002	0.37 (0.22, 0.63) <0.001
Model 2^b^	1.00	0.59 (0.43, 0.81)	0.59 (0.40, 0.88)	0.54 (0.35, 0.82)	0.014	0.46 (0.27, 0.78) 0.003
Model 3^c^	1.00	0.60 (0.44, 0.81)	0.59 (0.40, 0.89)	0.53 (0.35, 0.80)	0.010	0.45 (0.27, 0.76) 0.003

a Model 1 was adjusted for age (continuous), sex (men or women), and ethnicity (non-Hispanic white, non-Hispanic Black, Mexican American, and other race).

b Model 2 was adjusted for education (below high school, high school, and above high school), BMI (continuous), smoking status (never smoker, former smoker, and current smoker), drinking status (non-drinker, low-to-moderate drinker, and heavy drinker), recreational activity (inactive, moderately active, and vigorously active), and total energy intakes (in quartiles).

c Model 3 was further adjusted for blood pressure level (SBP/DBP ≥ 160/100 mmHg or SBP/DBP <160/100 mmHg), anti-hypertensive medicine use (yes or no), hyperlipidemia (yes or no), diabetes (yes or no), and CVD (yes or no).

### 3.3. Subgroup analysis

Consistent findings were found between HEI-2010 scores and all-cause mortality when stratifying the analysis by age (≤ 60 and >60 y), sex (male and female), ethnicity (non-Hispanic white and others), education (below/high school and above high school), BMI (<30 and ≥30), drinking status (non-drinker and drinker), smoking status (never smoker and former/current smoker), recreational activity (inactive and active), hyperlipidemia (no or yes), diabetes (no or yes), CVD (no or yes), and blood pressure level (SBP/DBP ≥ 160/100 mmHg or SBP/DBP <160/100 mmHg) (Table 3). Whereas, when the analysis was stratified by anti-hypertensive medicine use (yes or no), the subgroup dataset analyses were all statistically significant (*P*-trend <0.05), but the direction was not consistent across subgroups (Table 3). No significant interactions were found between HEI-2010 scores and strata variables (all *P* for interaction > 0.05), except for smoking (*P* for interaction= 0.022). Stronger inverse relationship between HEI-2010 scores and all-cause mortality in the hypertension population was observed in adults who never smoked.

**Table 3:** Stratified analyses of the associations between HEI-2010 scores and all-cause mortality among participants with hypertension.

**Characteristic**	**HEI-2010 scores HR (95% CI)** ^a^				***P*-trend**	***P*-interaction^b^**
	**Q1 14.0–44.1**	**Q2 44.1–53.9**	**Q3 53.9–64.1**	**Q4 64.1–98.8**		
Age, y						0.649
<60 (*n* = 3,205)	1.00	0.90 (0.59, 1.37)	0.83 (0.52, 1.33)	0.78 (0.44, 1.39)	0.338	
≥60 (*n* = 3,485)	1.00	0.83 (0.68, 1.00)	0.83 (0.65, 1.06)	0.73 (0.57, 0.93)	0.034	
Sex						0.090
Women (*n* = 3,471)	1.00	0.96 (0.73, 1.26)	0.82 (0.61, 1.10)	0.81 (0.58, 1.14)	0.161	
Men (*n* = 3,219)	1.00	0.75 (0.57, 0.98)	0.78 (0.62, 1.00)	0.59 (0.42, 0.83)	0.002	
Ethnicity						0.961
Non-Hispanic white (*n* = 2,922)	1.00	0.83 (0.68, 1.03)	0.76 (0.60, 0.97)	0.66 (0.49, 0.88)	0.003	
Others (*n* = 3,768)	1.00	0.76 (0.58, 0.98)	0.78 (0.57, 1.07)	0.69 (0.50, 0.94)	0.040	
Education						0.617
Below/high school (*n* = 3,611)	1.00	0.81 (0.66, 1.00)	0.87 (0.66, 1.13)	0.81 (0.62, 1.06)	0.216	
Above high school (*n* = 3,039)	1.00	0.88 (0.62, 1.23)	0.68 (0.46, 1.01)	0.56 (0.39, 0.82)	0.001	
BMI, kg/m^2^						0.793
<30 (*n* = 3,319)	1.00	0.97 (0.74, 1.27)	0.86 (0.64, 1.16)	0.70 (0.52, 0.93)	0.011	
≥30 (*n* = 3,371)	1.00	0.72 (0.56, 0.93)	0.66 (0.49, 0.88)	0.70 (0.49, 1.00)	0.043	
Recreational activity						0.126
Inactive (*n* = 4,060)	1.00	0.87 (0.72, 1.04)	0.87 (0.70, 1.01)	0.75 (0.59, 0.96)	0.025	
Active (*n* = 2,630)	1.00	0.69 (0.46, 1.03)	0.51 (0.34, 0.77)	0.50 (0.32, 0.77)	0.002	
Drinking status						0.660
Non-drinker (*n* = 2,671)	1.00	0.69 (0.54, 0.88)	0.70 (0.55, 0.88)	0.64 (0.49, 0.84)	0.003	
Drinker (*n* = 4,019)	1.00	0.97 (0.72, 1.31)	0.86 (0.62, 1.18)	0.67 (0.48, 0.92)	0.006	
Smoking status						0.022
Never (*n* = 3,452)	1.00	0.61 (0.46, 0.82)	0.70 (0.54, 0.92)	0.59 (0.43, 0.81)	0.0139	
Former/Current (*n* = 3,238)	1.00	0.93 (0.75, 1.16)	0.76 (0.59, 0.97)	0.62 (0.46, 0.84)	<0.001	
Hyperlipidemia						0.132
No (*n* = 1,331)	1.00	0.56 (0.34, 0.94)	0.82 (0.51, 1.31)	0.50 (0.30, 0.85)	0.036	
Yes (*n* = 5,359)	1.00	0.87 (0.69, 1.09)	0.75 (0.59, 0.96)	0.71 (0.54, 0.93)	0.007	
Diabetes						0.444
No (*n* = 4,611)	1.00	0.78 (0.61, 0.98)	0.72 (0.55, 0.94)	0.64 (0.48, 0.87)	0.007	
Yes (*n* = 2,079)	1.00	0.93 (0.65, 1.31)	0.90 (0.67, 1.20)	0.75 (0.51, 1.10)	0.100	
CVD						0.138
No (*n* = 5,410)	1.00	0.81 (0.66, 1.00)	0.68 (0.54, 0.85)	0.62 (0.47, 0.80)	<0.001	
Yes (*n* = 1,280)	1.00	0.86 (0.62, 1.17)	1.02 (0.75, 1.39)	0.84 (0.60, 1.19)	0.516	
Blood pressure level						0.910
SBP/DBP <160/100 (*n* = 5,976)	1.00	0.81 (0.67, 0.98)	0.79 (0.64, 0.98)	0.71 (0.56, 0.91)	0.007	
SBP/DBP ≥ 160/100 (*n* = 714)	1.00	0.88 (0.47, 1.67)	0.71 (0.42, 1.22)	0.50 (0.28, 0.91)	0.011	
Anti-hypertensive drug use						0.664
No (*n =* 5,579)	1.00	0.91 (0.72, 1.15)	0.84 (0.67, 1.06)	0.72 (0.54, 0.95)	0.012	
Yes (*n* = 1,111)	1.00	0.47 (0.29, 0.76)	0.52 (0.34, 0.80)	0.72 (0.54, 0.95)	0.035	

a Data are expressed as HRs (95% CIs) adjusted for age (continuous), sex (men or women), ethnicity (non-Hispanic white, non-Hispanic Black, Mexican American, and other race), education (below high school, high school, and above high school), BMI (continuous), smoking status (never smokers, former smoker, and current smokers), drinking status (non-drinker, low-to-moderate drinker, and heavy drinker), recreational activity (inactive, moderately active, and vigorously active), total energy intakes (in quartiles), blood pressure level (SBP/DBP ≥ 160/100 mmHg or SBP/DBP <160/100 mmHg), anti-hypertensive medicine use (yes or no), hyperlipidemia (yes or no), diabetes (yes or no), and CVD (yes or no). We did not include the strata variable in the model when stratifying by itself.

b The interaction between continuous HEI-2010 scores and stratification variables was examined using the Wald test.

### 3.4. HEI-2010 component scores and mortality

**Figure 2:**
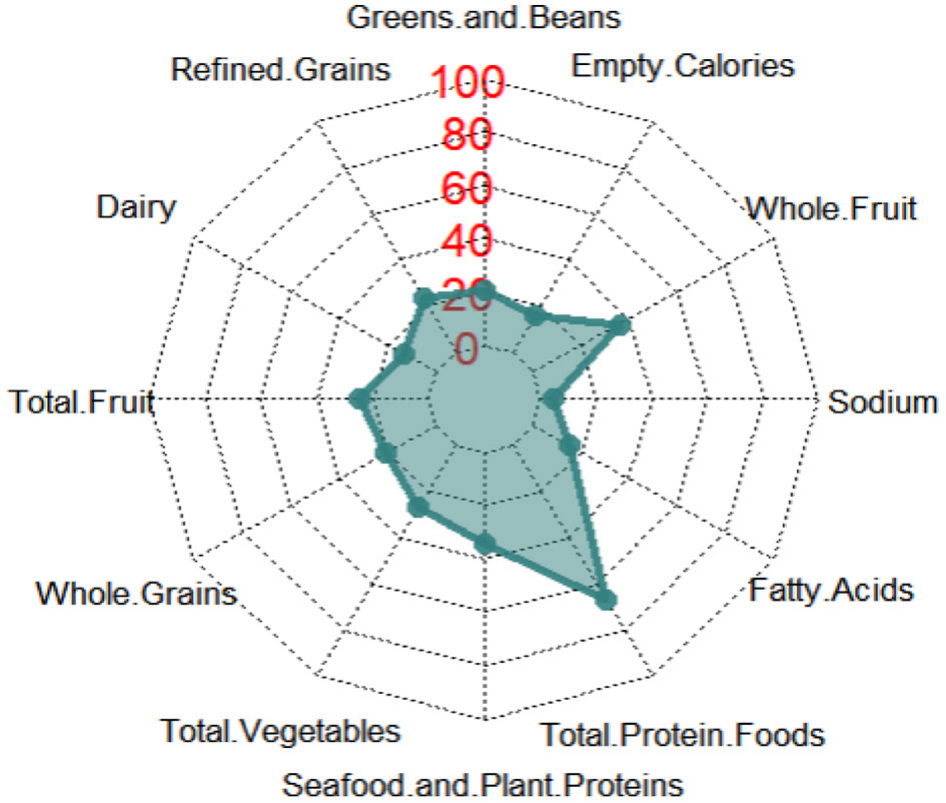
Radar graph depicting the weighted proportions of participants who obtained the highest component score for each component of HEI-2010. The radar graph shows all 12 components of HEI-2010, including total vegetables, greens and beans, total fruits, whole fruits, total dairy, total protein foods, seafood and plant proteins, whole grains and fatty acid ratio, sodium, refined grains, and empty calories.

There were significant differences in the weighted proportions of participants who obtained the maximum component score by the HEI-2010 component (Table 4), which we presented as a radar plot (Figure 2). The proportion of participants receiving the highest component score for each component was over 60% for total protein foods, 20–40% for total seafood and plant proteins, greens and beans, vegetables, refined grains, total fruits, and whole fruits; 10–20% for dairy, unsaturated fatty acids, and empty calories; and <10% for sodium and whole grains. When the components of HEI-2010 were evaluated (Table 4), higher component scores were linked to a lower all-cause mortality risk in all three multivariate models for six of the 12 components: seafood and plant protein, total protein foods, greens and beans, total vegetables, unsaturated fatty acids, and empty calories, reducing the risk of all-cause mortality by 21–29%. For instance, participants consuming at least 2.5 oz per 1,000 calories a day of total protein foods reduced their risk of death from all causes by 25% based on the results in Model 3. Other HEI-2010 components not shown to be linked to all-cause mortality were whole grains, refined grains, dairy, sodium, whole fruit, and total fruit.

**Table 4:** HR for all-cause mortality according to HEI-2010 components scores among participants with hypertension in NHANES 2007–2014^a^.

**HEI 2010 components**	**Receive max score^b^, %**	**Model 1 HR (95% CI)**	**Model 2 HR (95% CI)**	**Model 3 HR (95% CI)**
**Adequacy**				
Total fruit^**^	25.12 (23.24,27.00)	0.73 (0.58,0.93)^+^	1.07 (0.85,1.35)	0.98 (0.78,1.24)
Whole fruit^**^	35.90 (33.15,38.64)	0.67 (0.55,0.80)^+^	0.90 (0.74,1.11)	0.88 (0.72,1.08)
Total vegetables^***^	27.03 (24.71,29.34)	0.64 (0.53,0.79)^+^	0.71 (0.58,0.88)^+^	0.71 (0.57,0.88)^+^
Greens and beans^**^	20.84 (19.03,22.65)	0.67 (0.55,0.80)^+^	0.76 (0.63,0.92)^+^	0.77 (0.63,0.94)^+^
Whole grains^****^	6.39 (5.63,7.15)	0.79 (0.66,0.94)^+^	0.94 (0.78,1.13)	0.91 (0.76,1.09)
Dairy^***^	12.95 (11.42,14.48)	0.99 (0.79,1.24)	1.02 (0.82,1.27)	1.04 (0.83,1.29)
Total protein foods^*^	67.14 (62.63,71.65)	0.75 (0.65,0.87)^+^	0.75 (0.66,0.86)^+^	0.75 (0.66,0.85)^+^
Seafood and plant proteins^**^	34.00 (31.27,36.72)	0.60 (0.49,0.74)^+^	0.75 (0.61,0.93)^+^	0.76 (0.62,0.94)^+^
Fatty acids^*****^	15.25 (13.80,16.71)	0.78 (0.68,0.90)^+^1	0.81 (0.69,0.95)^+^	0.79 (0.68,0.93)^+^
**Moderation**				
Refined grains^***^	23.77 (21.46,26.08)	0.79 (0.66,0.94)^+^	0.94 (0.78,1.13)	0.91 (0.76,1.09)
Sodium^****^	5.12 (4.46,5.78)	0.96 (0.82,1.14)	1.09 (0.90,1.32)	1.10 (0.92,1.33)
Empty calories^******^	16.43 (15.06,17.80)	0.71 (0.59,0.85)^+^	0.78 (0.64,0.94)^+^	0.75 (0.62,0.90)^+^

^a^Data are expressed as HRs (95% CIs) adjusted for age (continuous), sex (men or women), ethnicity (non-Hispanic white, non-Hispanic Black, Mexican American, and other race), education (below high school, high school, and above high school), BMI (continuous), smoking status (never smokers, former smokers, and current smokers), drinking status (non-drinker, low-to-moderate drinker, and heavy drinker), recreational activity (inactive, moderately active, and vigorously active), total energy intakes (in quartiles), blood pressure level (SBP/DBP ≥ 160/100 mmHg or SBP/DBP <160/100 mmHg), anti-hypertensive medicine use (yes or no), hyperlipidemia (yes or no), diabetes (yes or no), and CVD (yes or no).

^b^Weighted proportions of participants who obtained the maximum component score for each component of HEI-2010.

^*^Those with a maximum score of 5, 10, or 20 compared to those with a score below it (e.g., max vs. <max).

^**^Those with a maximum score of 5, 10, or 20 compared to those with a score of zero (e.g., max vs. zero).

^***^Those with a maximum score of 5, 10, or 20 compared to those with a score below the 25th percentile (e.g., max vs. below the 25th percentile).

^****^Those with above minimum score compared to those with a score of zero (e.g., min <vs. zero).

^*****^Those with a score above the 50th percentile compared to those with a score below the 50th percentile.

^******^Those with a score above the 75th percentile compared to those with a score below the 25th percentile.

+ P-value <0.05.

### 3.5. Sensitivity analyses

In sensitivity analyses, the inverse relationship of HEI-2010 scores with heart disease and all-cause mortality remained largely unchanged after excluding the participants who died within the first 2 years of follow-up (*n* = 199) (Supplementary Table 2). After additional adjustments for dietary intakes of cholesterol, fiber, total fat, vitamin E, vitamin A, vitamin C, and biomarkers of serum total cholesterol, eGFR, the results remained largely unchanged (Supplementary Table 3). Finally, HEI-2010 scores were not related to cerebrovascular mortality and cancer mortality (Supplementary Table 4).

## 4. Discussion

To date, it is the first research to examine the relationship of the HEI-2010 and its components with mortality from heart disease and all causes in adults with hypertension. We observed that higher HEI-2010 scores were linked to lower heart disease mortality and all-cause mortality risk independent of various factors, which included lifestyle and dietary factors, anti-hypertensive medicine use, and blood pressure levels. We demonstrated the robustness of our findings through stratified analyses and sensitivity analyses. According to our study, among the 12 components of HEI-2010, a higher intake of greens and beans, seafood and plant proteins, total protein foods, vegetables, and unsaturated fatty acids, as well as moderate consumption of empty calories, were linked to a lower risk of all-cause mortality.

Previous studies including some meta-analysis studies have investigated the associations of healthy dietary patterns with cardiovascular and all-cause mortality and reached consistent findings with ours. They indicated that the inverse relationships of healthy dietary patterns with cardiovascular and all-cause mortality were statistically significant in both general populations and other people (11, 12, 17–21). For instance, according to a linear dose-response meta-analysis, each 5-point increment in compliance with Dietary Approaches to Stop Hypertension (DASH) significantly reduced the all-cause mortality risk (assessed in 13 cohort studies, 9 publications, including 1,240,308 participants) and cardiovascular mortality (assessed in 12 cohorts, 9 publications, including 1,314,675 participants) for 5% (6–4%) and 4% (5–2%), respectively (20). In addition, in a prospective multiethnic cohort study, each healthy dietary pattern including HEI-2010, the Alternative Healthy Eating Index (AHEI-2010), DASH, and the Alternate Mediterranean Diet was linked to reduced risk of deaths from cardiovascular disease and all causes (11). The same results were also seen in two other prospective cohort studies that recruited older adults (12) and postmenopausal women in the United States (US) (18), respectively. One possible explanation for the consistent findings is that despite there being multiple dietary pattern index scores, they tend to converge in preventing major chronic diseases, such as diabetes (22, 23) and cardiovascular disease (24, 25), as they are derived from many of the same core principles emphasizing vegetables, whole grains, plant-based proteins, and fruits, food combinations that are primarily rich in antioxidants and anti-inflammatory nutrients. A systematic review with 16 observational and 13 interventional studies indicated an inverse relationship between healthy dietary patterns with oxidative stress and pro-inflammatory biomarkers (26). In addition, healthy dietary patterns may improve lipid metabolism, blood pressure, and endothelial function, and has anti-oxidative and anti-inflammatory properties (25, 27).

However, among adults with hypertension who tend to have endothelial dysfunction, increased oxidative stress, pro-inflammatory release, insensitivity to vasodilators, arterial vascular smooth stiffening (14, 15), and with a higher incidence of CVD and all-cause mortality, it is still not well known whether HEI-2010 has a long-term effect on mortality in this specific population. Although previous reviews and studies have reported beneficial effects on blood pressure control with HEI-2010 (8). Based on a nationally representative American adult sample with a long follow-up duration, the current study found inverse relationships between HEI-2010 and mortality from heart disease and all causes in adults with hypertension. Our results were consistent with a study performed on male diabetic physicians, where diabetes is characterized by increased pro-inflammatory and oxidative status. In this prospective cohort study of 1,163 male physicians with diabetes only, an inverse relationship between the AHEI-2010 score and all-cause mortality was found (HR = 0.59; 95% CI 0.44, 0.79) (21). A further analysis of the 12 individual components of HEI-2010 was conducted to determine possible dietary components that might affect all-cause mortality. It found that a higher intake of greens and beans, seafood and plant protein, total vegetables, total protein foods, unsaturated fatty acids, and moderate consumption of empty calories were associated with a 21–29% reduction in the all-cause mortality risk in adults with hypertension. The results can be explained as follows.

First, vegetables are rich in different nutrients and bioactive compounds, such as phytochemicals, vitamins, minerals, and fibers, which have cardioprotective effects, including anti-inflammation, anti-oxidation, anti-platelet properties, regulate blood pressure and lipid metabolism, improve endothelial function, and reduce myocardial injury (28, 29). As reported in one review, nitrate, which is rich in vegetables, is now considered a critical bioactive phytochemical with cardioprotective performances, increasing nitrogen oxide (NO) and other nitrogen oxides *via* the nitrate-nitrite-nitric oxide pathway to improve endothelial function, lower blood pressure, regulate arterial stiffness, reduce ischemia-reperfusion injury, modulate blood flow, and anti-platelet aggregation (30). In addition, vegetables are a significant source of potassium, and higher potassium intake was related to lower blood pressure, especially a high potassium/sodium ratio (31). Third, green vegetable soya bean is rich in carbohydrates, omega-3 fatty acids, protein, fiber, and a variety of micronutrients providing nutrients for humans, where steroids 7, saponins 2, alkaloids 6, and isoflavones 5 are found, exhibiting anti-oxidative and anti-inflammatory properties to some extent (32, 33). Fourth, the fatty acids ratio is expressed as monounsaturated and polyunsaturated fatty acids (PUFA) to saturated fatty acids. Omega-3 PUFA has been reported to reduce cardiovascular disease risk by regulating lipid metabolism and platelet aggregation (34). Also, seafood is a major source of omega-3 PUFA, which has been discovered to provide anti-inflammatory effects (35) and regulate blood lipids (36). Fifth, plant protein consumption has been found to lower the levels of total cholesterol, LDL cholesterol, and blood pressure (37, 38). Finally, in the HEI-2010, empty calories are those derived from added sugars and solid fats (7), there has been an association between solid fat intake as well as added sugar intake with mortality in previous studies (39, 40). Excessive added sugars intake is associated with elevated triglyceride levels (41) and inflammatory markers (42), which are crucial determinants in the pathogenesis of CVD. Therefore, it is necessary to limit the intake of added sugar. Through the mechanisms described earlier, the HEI-2010 may improve cardiovascular conditions and reduce all-cause mortality in adults with hypertension.

Furthermore, in adults with hypertension, HEI-2010 seemed to have a stronger protective effect against mortality events in non-smokers than in smokers. The finding may be due to the fact that smoking increases cardiovascular mortality and morbidity, which is supported by epidemiological studies (43, 44). When the analysis was stratified by anti-hypertensive medicine use (yes or no), the subgroup dataset analyses were all statistically significant, but the direction was not consistent across subgroups. The most common reason may be that there was an interaction between subgroup factors or between the observed subgroup factors with unknown factors. Positive results found in subgroup analyses have an extremely high probability of being false positive due to the fact that the subgroup analyses may not maintain randomization within subgroups, have an insufficient sample size, and low degree of certainty. Therefore, the essence of the evidence is a result of an observational study, which needs to be interpreted with caution and applied carefully to guide the work of clinical practice.

This study has several strengths. First, it is the first study to assess the relationship between the HEI-2010 and mortality from heart disease and all causes in adults with hypertension based on NHANES. Second, this study used data from NHANES, which collected and reported data using standardized procedures and strict quality assurance. Third, we were able to generalize our results to the US adult population by utilizing a broad, nationally representative database to estimate diet quality. Finally, modeled upon the 2010 DGA recommendations, the HEI-2010 is a reliable and valid measurement of dietary quality for Americans.

There are also some potential limitations to the current study. First, the current study did not have detailed information on the severity of hypertension, although the results did not change significantly after further adjustment of blood pressure levels and anti-hypertensive medicine use. In addition, as some missing data in the database, we cannot include all potentially significant variables, for example, more than 50% of the data for CRP and LDL cholesterol were missing.

## 5. Conclusion

According to this prospective cohort study of adults with hypertension, we observed an inverse association between HEI-2010 and mortality from heart disease and all causes. Based on the findings, it may be helpful to guide the dietary intake of adults with hypertension. Further studies are needed to support these results.

## Data availability statement

Publicly available datasets were analyzed in this study. This data can be found here: https://www.cdc.gov/nchs/nhanes/index.htm.

## Ethics statement

The studies involving human participants were reviewed and approved by National Center for Health Statistics (NCHS) Research Ethics Review Board. The patients/participants provided their written informed consent to participate in this study. Written informed consent was obtained from the individual(s) for the publication of any potentially identifiable images or data included in this article.

## Author contributions

Study design: YZ, DL, and HZ. Data collection and analysis: YZ and DL. Writing the manuscript: YZ. All authors have read and agreed to the published version of the manuscript.
