# Supplementary material for: Associations of the Healthy Eating Index-2010 with risk of all-cause and heart disease mortality among adults with hypertension: Results from the National Health and Nutrition Examination Survey 2007–2014
Source: Front Nutr. 2023 Mar 3;10:1077896. doi: 10.3389/fnut.2023.1077896 (PMC10020655; doi:10.3389/fnut.2023.1077896)
Supplement: Supplementary file 1 [file Data_Sheet_1.docx]

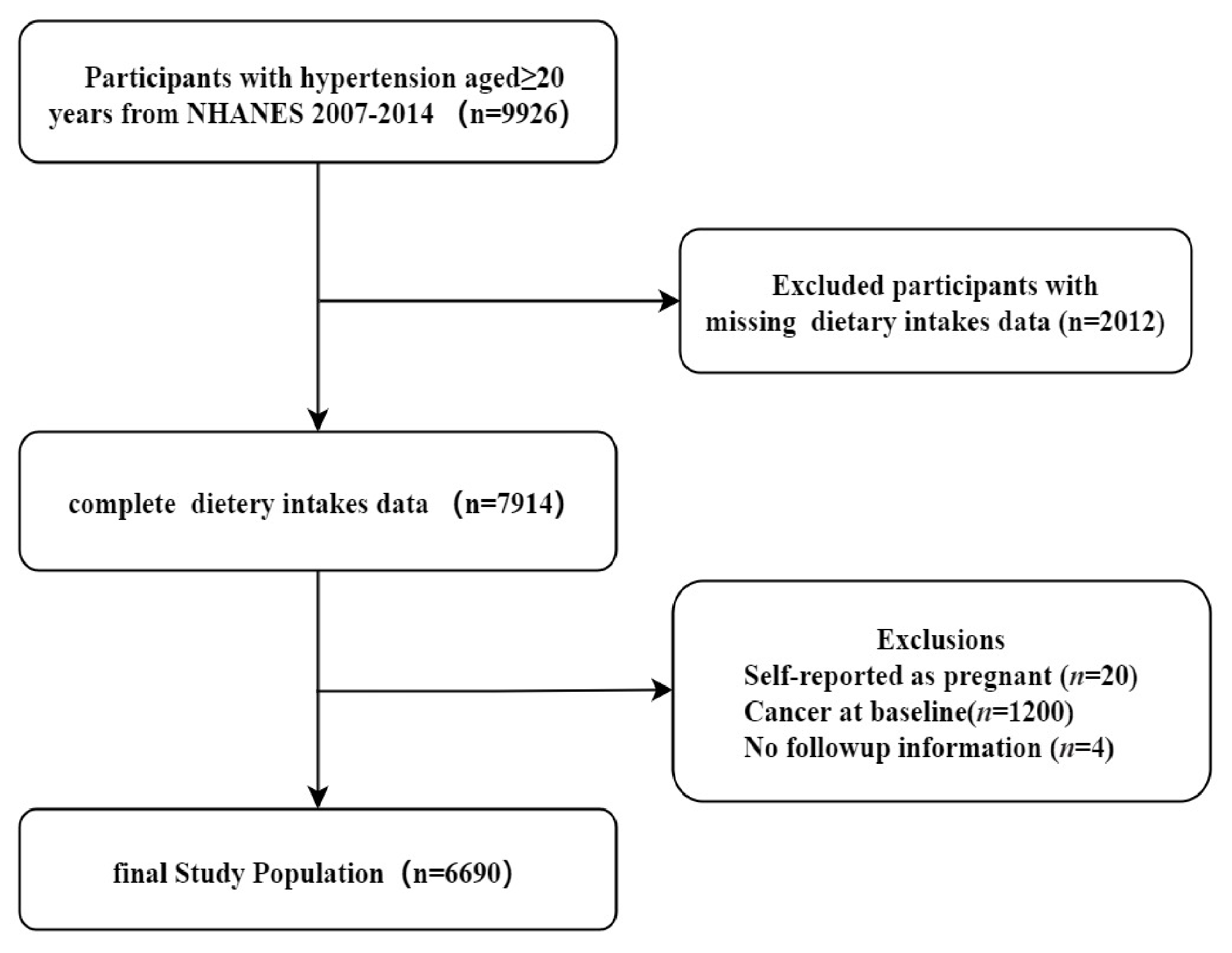


**Supplementary** **Figure 1.**Flowchart of the study participants.Based on the NHANES 2007–2014 surveys,9926 participants with hypertension aged 20 and older years were included,and then 2012 participants with missing dietary intake data were excluded.Those reporting pregnancy (n=20), cancer (n=1200), or with no follow-up data (n=4) were further excluded, with a final inclusion of 6690 hypertensive participants.

**Supplemental Table 1.**Healthy Eating Index-2010 components and standards for scoring

| Components | Maximum points | Standard for maximum score | Standard for minimum score of zero |
| --- | --- | --- | --- |
| **Adequacy:** |  |  |  |
| Total Fruit ^a^ | 5 | ≥0.8 cup equivalent per 1,000 kcal | No Fruit |
| Whole Fruit ^b^ | 5 | ≥ 0.4 cup equivalent per 1,000 kcal | No Whole Fruit |
| Total Vegetables ^c^ | 5 | ≥ 1.1 cup equivalents per 1,000 kcal | No Vegetables |
| Greens and Beans ^c^ | 5 | ≥ 0.2 cup equivalent per 1,000 kcal | No Dark Green Vegetables  or Beans and Peas |
| Whole Grains | 10 | ≥ 1.5 oz equivalents per 1,000 kcal | No Whole Grains |
| Dairy ^d^ | 10 | ≥ 1.3 cup equivalents per 1,000 kcal | No Dairy |
| Total Protein Foods ^e^ | 5 | ≥2.5 oz equivalents per 1,000 kcal | No Protein Foods |
| Seafood and Plant Proteins ^e f^ | 5 | ≥0.8 oz equivalent per 1,000 kcal | No Seafood or Plant Proteins |
| Fatty Acids^g^ | 10 | (PUFAs+ MUFAs)/SFAs≥ 2.5 | (PUFAs+ MUFAs)/SFAs≤1.2 |
| **Moderation:** |  |  |  |
| Refined Grains | 10 | ≤1.8 oz equivalents per 1,000 kcal | ≥ 4.3 oz equivalents per 1,000 kcal |
| Sodium | 10 | ≤1.1 g per 1,000 kcal | ≥2.0 g per 1,000 kcal |
| Empty Calories ^h^ | 20 | ≤19% of energy | ≥50% of energy |

a Includes 100% fruit juice

b Includes all forms except juice.

c Includes any beans and peas not counted as Total Protein Foods .

d Includes all milk products, such as fluid milk, yogurt, and cheese, and fortified soy beverages.

e Beans and peas are included here (and not with vegetables) when the Total Protein Foods standard is otherwise not met.

f Includes seafood, nuts, seeds, soy products (other than beverages) as well as beans and peas counted as Total Protein Foods.

g Ratio of polyunsaturated fatty acids (PUFAs) and monounsaturated fatty acids (MUFAs) to saturated fatty acids (SFAs).

h Calories from solid fats, alcohol, and added sugars; threshold for counting alcohol is 13 g/1,000 kcal.

**Supplemental Table 2.**HR of all cause and heart disease mortality according to [quartiles](javascript:;) of HEI-2010 scores among participants with hypertension after excluding participants who died within two years of follow-up in NHANES 2007–2014 (n=6591)

|  | HEI 2010scores HR (95% CI) P-value | | | |  |
| --- | --- | --- | --- | --- | --- |
| Characteristic | Q1 | Q2 | Q3 | Q4 | *P*-trend |
| range | 14.0-44.1 | 44.1-54.0 | 54.0-64.2 | 64.2-98.8 |  |
| all cause mortality |  |  |  |  |  |
| No. deaths/total | 267/1615 | 267/1633 | 279/1620 | 247/1623 |  |
| Model 1^1^ | 1.00 | 0.70(0.57,0.85) | 0.65(0.53,0.80) | 0.51(0.41,0.65) | <0.001 |
| Model 2^2^ | 1.00 | 0.79(0.64,0.97) | 0.80(0.65,0.99) | 0.71(0.55,0.90) | 0.010 |
| Model 3^3^ | 1.00 | 0.79(0.65,0.97) | 0.80(0.65,0.99) | 0.68(0.53,0.88) | 0.005 |
| heart disease mortality |  |  |  |  |  |
| No. deaths/total | 70/1615 | 66/1633 | 73/1620 | 75/1623 |  |
| Model 1^1^ | 1.00 | 0.54(0.38,0.75) | 0.53(0.35,0.81) | 0.48(0.31,0.76) | 0.010 |
| Model 2^2^ | 1.00 | 0.59(0.43,0.82) | 0.61(0.39,0.94) | 0.57(0.37,0.88) | 0.044 |
| Model 3^3^ | 1.00 | 0.60(0.44,0.82) | 0.61(0.39,0.95) | 0.55(0.35,0.87) | 0.036 |

1Model1 was adjusted for age (continuous), sex (male or female), and ethnicity (non-Hispanic White, non-Hispanic Black,Mexican American and other race).

2Model 2 was further adjusted for education (below high school, high school, and above high school),BMI (continuous), drinking status (nondrinker, low to moderate drinker, heavy drinker), smoking status(never smokers, former smokers, current smokers),recreational activity(inactive group, moderate active group, vigorous active group), total energy intakes (in [quartiles](javascript:;)).

3Model 3 was further adjusted for blood pressure level ( SBP/DBP≥160/100mmHg or SBP/DBP<160/100mmHg), anti-hypertensive medicine use (yes or no), [hyperlipidemia](javascript:;)(yes or no), diabetes (yes or no),and CVD (yes or no).

**Supplemental Table 3.**HR of all cause mortality according to [quartiles](javascript:;) of HEI-2010 scores among participants with hypertension with further adjustment of dietary factors and serum biomarkers in NHANES 2007–2014.

|  | HEI 2010 scores HR (95% CI) P-value | | | |  |
| --- | --- | --- | --- | --- | --- |
| Characteristic | Q 1 | Q 2 | Q3 | Q 4 | P-trend |
| range | 14.0-44.1 | 44.1-53.9 | 53.9-64.1 | 64.1-98.8 |  |
| No. deaths/total | 320/1668 | 329/1682 | 323/1667 | 287/1673 |  |
| Model 1* | 1.00 | 0.82(0.70,0.97) | 0.78(0.64,0.95) | 0.68(0.54,0.85) | 0.001 |
| Model 1+dietary factors** | 1.00 | 0.82(0.69,0.98) | 0.77(0.62,0.96) | 0.64(0.49,0.83) | 0.001 |
| Model 1+ biomarkers*** | 1.00 | 0.83(0.70,1.00) | 0.77(0.62,0.95) | 0.63(0.49,0.82) | 0.001 |

*Cox proportional hazards models were used to estimate the HRs (95% CIs) of all-cause mortality according to [quartiles](javascript:;) of HEI-2010 scores. Results were adjusted for age (continuous), sex (male or female), and ethnicity (non-Hispanic White, non-Hispanic Black,Mexican American and other race), education (below high school, high school, and above high school),BMI (continuous), drinking status (nondrinker, low to moderate drinker, heavy drinker), smoking status(never smokers, former smokers, current smokers),recreational activity(inactive group, moderate active group, vigorous active group), total energy intakes (in [quartiles](javascript:;) ), blood pressure level (SBP/DBP≥160/100mmHg or SBP/DBP<160/100mmHg), anti-hypertensive medicine use (yes or no), [hyperlipidemia](javascript:;)(yes or no), diabetes (yes or no),and CVD (yes or no).

**Further adjusted for intakes of total fat, cholesterol, fiber,vitamin A,vitamin E,vitamin C (all in [quartiles](javascript:;)).

***Further adjusted for serum total cholesterol levels and eGFR(all in [quartiles](javascript:;)).

**Supplemental Table 4.** HR for cancer and cerebrovascular mortality according to HEI-2010 scores among participants with hypertension in NHANES 2007-2014

|  | HEI 2010 scores HR (95% CI) | | | |  |
| --- | --- | --- | --- | --- | --- |
| Characteristic | Quartile 1 | Quartile 2 | Quartile 3 | Quartile 4 | P-trend |
| range | 14.0-44.1 | 44.1-53.9 | 53.9-64.1 | 64.1-98.8 |  |
| cancer mortality |  |  |  |  |  |
| No. deaths/total | 57/1668 | 70/1682 | 61/1667 | 51/1673 |  |
| Model 1^1^ | 1.00 | 0.94(0.57,1.57) | 0.86(0.55,1.34) | 0.67(0.41,1.10) | 0.085 |
| Model 2^2^ | 1.00 | 1.13(0.66,1.94) | 1.14(0.69,1.87) | 0.92(0.52,1.64) | 0.751 |
| Model 3^3^ | 1.00 | 1.11(0.64,1.92) | 1.14(0.70,1.86) | 0.89(0.50,1.59) | 0.672 |
| cerebrovascular mortality |  |  |  |  |  |
| No. deaths/total | 13/1668 | 26/1682 | 21/1667 | 22/1673 |  |
| Model 1^1^ | 1.00 | 1.99(0.90,4.40) | 1.10(0.48,2.48) | 1.19(0.52,2.73) | 0.556 |
| Model 2^2^ | 1.00 | 2.11(0.97,4.63) | 1.25(0.55,2.85) | 1.52(0.65,3.57) | 0.867 |
| Model 3^3^ | 1.00 | 2.11(0.96,4.66) | 1.26(0.54,2.93) | 1.47(0.62,3.48) | 0.950 |

1Model1 was adjusted for age (continuous), sex (male or female), and ethnicity (non-Hispanic White, non-Hispanic Black, Mexican American and other race).

2Model 2 was further adjusted for education (below high school, high school, and above high school),BMI (continuous), drinking status (nondrinker, low to moderate drinker, heavy drinker), smoking status(never smokers, former smokers, current smokers), recreational activity(inactive group, moderate active group, vigorous active group), total energy intakes (in [quartiles](javascript:;)).

3Model3 was further adjusted for blood pressure level (SBP/DBP≥160/100mmHg or SBP/DBP<160/100mmHg),anti-hypertensive medicine use (yes or no),[hyperlipidemia](javascript:;) (yes or no), diabetes (yes or no),and CVD (yes or no).
